# A cobalt(ii) chain based on pymca generated *in situ* from the hydrolysis of 2-cyanopyrimidine: spin canting and magnetic relaxation†

**DOI:** 10.1039/c9ra05354a

**Published:** 2019-10-02

**Authors:** Jie Zhang, Qian-Nan Zhao, Feng Yang, Lei Yin, Miao-Miao Li, Zhenxing Wang, Zhongwen Ouyang, Zheng-Cai Xia, Tuo-Ping Hu

**Affiliations:** Department of Chemistry, College of Science, North University of China Xueyuan Road 3 Taiyuan 030051 P. R. China hutuoping@nuc.edu.cn; Wuhan National High Magnetic Field Center, School of Physics, Huazhong University of Science and Technology Wuhan 430074 P. R. China xia9020@hust.edu.cn

## Abstract

A one-dimensional (1D) coordination polymer, [{Co_2_(pymca)_2_·(H_2_O)_4_}SO_4_·2H_2_O]_*n*_ (1) (pymca = 2-carboxypyrimidine), was solvothermally synthesized *via* the reaction of 2-cyanopyrimidine and Co(SCN)_2_. A bidentate pymca ligand was formed *in situ* by the hydrolysis of 2-cyanopyrimidine. Furthermore, in this study, the magnetic properties of complex 1 were investigated in detail. The results indicated that complex 1 showed a single-chain magnet (SCM) behavior below *ca.* 3 K. The energy barrier (Δ*τ*_1_/*k*_B_) and preexponential factor (*τ*_0_) of SCM were 31.2 K and 5.4 × 10^−9^ s, respectively.

## Abstract 2

A cobalt(ii) chain based on pymca generated *in situ* from the hydrolysis of 2-cyanopyrimidine. The magnetic properties indicate that complex 1 shows the single-chain magnet (SCM) behavior with the energy barrier of 31.2 K.

## Graphical abstract

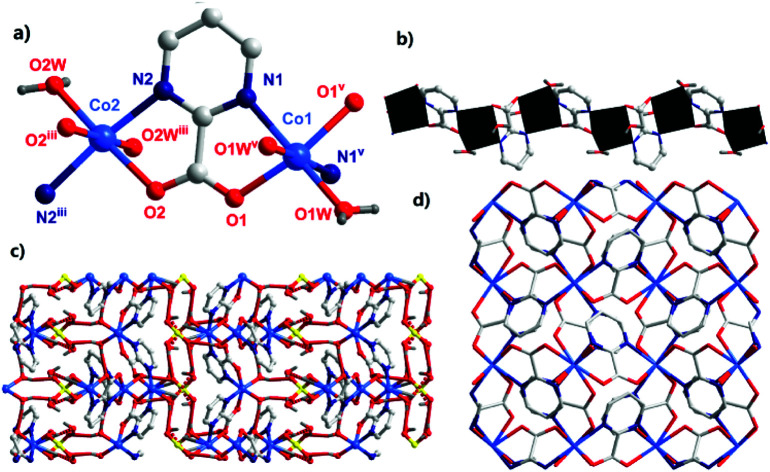

## Introduction

Molecule-based magnetic materials with structural diversity have a number of advantages, such as their controllable dimensions, and low energy consumption, which have attracted extensive attention.^1–4^ Among them, single-chain magnets (SCMs), one type of low-dimensional molecule-based magnetic materials, have many kinds of fundamental physical properties^5,6^ such as finite-sized effects and a slow relaxation behavior for the magnetization. Since the first report on SCMs in 2001,^7^ the molecular chain system has received considerable attention due to its potential application and value in molecular spintronic and information storage,^8–11^ creating a new trend in the field of molecular magnetism. In the previous design and synthesis of SCMs, researchers have greatly desired to eliminate the interactions between the chains in order to obtain an isolated ferromagnetic, ferrimagnetic or spin-canted Ising chain.^7,12^ However, previous studies have shown that intermolecular interactions still preserve the magnetic relaxation produced by the SCM component while forming three-dimensional ordering. A typical example is that by Ishida *et al.*^5^ and they first observed a SCM behavior below the 3D ordering temperature (*T*_C_) in 2008. From then until now, the magnetic relaxation of the SCM component in antiferromagnetic or metamagnetic phases was discovered in many systems,^13–19^ which indicated that the weak magnetic interactions between the chains was perhaps not the most important prerequisite for observing the slow relaxation behavior of magnetization. Therefore, low-dimensional magnets beyond the Glauber theory^20^ might result in the new development of molecule-based magnets, particularly for SCMs.

It is well-known that the Co^II^ ion has strong Ising-like magnetic anisotropy and easily creates an energy barrier for the magnetization reversal. Therefore, it is always used as the preferred spin carrier.^21–27^ 2-Carboxypyrimidine (pymca)^28^ shows a similar coordination mode to oxalate^29^ and oxamide. Controlling the ratio of metal ions and ligands or changing the polarity of the solvent may reduce the number of bridging ligands around one metal ion, further leading to a one-dimensional (1D) structure^30^ with the relaxed magnetization. Inspired by the above information, we successfully synthesized a 1D complex, {[Co_2_(pymca)_2_·(H_2_O)_4_]SO_4_·2H_2_O}_*n*_ (1), which exhibited a SCM behavior below 3 K.

## Experimental

### Reagents and general procedures

All reagents and solvents were available for purchase and were used directly without further purification.

### Synthesis of {[Co_2_(pymca)_2_·(H_2_O)_4_] SO_4_·2H_2_O}_*n*_ (1)

(a) A mixture of Co(SCN)_2_ (0.5 mmol, 87.6 mg) and 2-cyanopyrimidine (0.5 mmol, 52.6 mg) was dissolved in 14 mL of a H_2_O/CH_3_CN (v : v = 1 : 1) mixed solution. The mixture was sealed in a 20 mL heavy-wall pressure resistant vessel, heated to 120 °C for 48 h, and then gradually cooled to 25 °C. Orange flaky crystals were collected with a yield of 56% (based on Co). Elemental analysis calcd (%) for C_10_H_18_Co_2_N_4_O_14_S: C (21.12), N (9.86), H (3.17), S (5.63); found: C (21.57), N (10.10), H (3.144), S (6.18). IR data (cm^−1^): 3546 s, 3474 s, 3420 s, 2398 m, 2282 m, 1626 s, 1582 s, 1474 m, 1402 s, 1296 w, 1152 m, 1106 m, 1034 m, 974 w, 874 w, 704 m, 676 m, 614 m.

(b) Because SO_4_^2−^ ions were found in the structure of complex 1, the synthesis of complex 1 was performed again. The reaction conditions were the same except that CoSO_4_·7H_2_O (0.5 mmol, 0.1406 g) was substituted for Co(SCN)_2_. Orange crystals were obtained with a yield of 60% (based on Co).

### X-ray crystallography

The X-ray diffraction data for complex 1 was ascertained by selecting a suitable size for the crystals and collection using a Bruker Smart CCD area-detector diffractometer with Mo-Kα radiation (*λ* = 0.71073 Å) using a *ω* scan mode at 296(2) K. The crystal structure was solved by the direct method and refined by full-matrix least-squares using the SHELXTL package. All of the non-hydrogen atoms were located by the Patterson's method^31*a*^ using the SHELXS program in the SHELXTL package and by subsequent difference Fourier syntheses.^31*b-c*^ All the hydrogens attached to the carbon were determined theoretically and refined with isotropic thermal parameters riding on their parents. All calculations were performed by SHELXTL-2018. Crystallographic data are given in Table S1.† Bond lengths and angles for 1 were selected and are listed in Table S2.†

### Physical measurements

Using the Nexus 870 FT-IR spectrometer, IR spectroscopy was performed with KBr particles in the range of 400–4000 cm^−1^. The elemental analyses of C, H, and N were recorded on a PerkinElmer 240C elemental analyzer. TGA was measured from 30 to 800 °C on a NETZSCH STA 449F3 analyzer at a heating rate of 5 °C min^−1^ under a N_2_ atmosphere. The ac magnetic susceptibility data were collected using a MPMS-XL7 Squid magnetometer (test conditions: *H*_ac_ = 5 Oe, *H*_dc_ = 0 Oe, frequencies ranging from 1 to 1488 Hz). The dc magnetic susceptibility data were measured in the temperature range of 1.8–300 K. The experimental susceptibilities were corrected for the diamagnetism of the samples as estimated from Pascal's tables^31*d*^ and the sample holder by a previous calibration. Pulsed-high-field magnetization measurements were performed at 1.9 K using a home-made pulsed field up to 30 T at the Wuhan National High Magnetic Field Center, P. R. China. The sweeping rate for the magnetic field was 5.0 kT s^−1^ on an average. The low-field magnetization data (on SQUID) were used to calibrate the high-field magnetization curve, which is a standard way to process the high field magnetization data.^31*e*^

## Results and discussion

### Synthesis

**Scheme 1:**
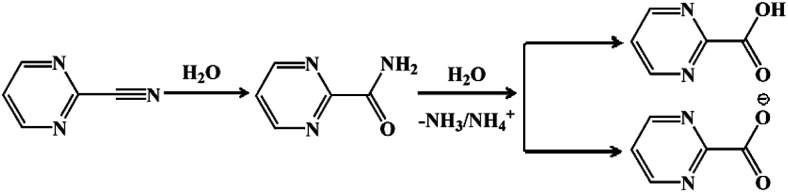
The hydrolysis process for 2-cyanopyrimidine to pymca.

The reaction for the hydrolysis of nitriles to carboxylic acids and ammonia is very common. The reaction temperature as well as the acidity and alkalinity of the solution will greatly affect the reaction rate.^28^ The mechanism for pymca generated from 2-cyanopyrimidine *in situ* is described in Scheme 1. Briefly, 2-cyanopyrimidine was hydrolyzed at a high temperature to form 2-amidopyrimidine, which lost ammonia or the ammonium ion under weakly basic conditions, finally forming 2-carboxypyrimidine or the 2-carboxypyrimidine ion. Moreover, a detailed discussion on the ligand pymca *in situ* synthesis by the hydrothermal reaction was described by Colacio *et al.*^28^ They believed pymca, a proposed intermediate between bipyrimidine and oxalate, was generated *in situ* from the hydrolysis of 2-cyanopyrimidine. Herein, we only briefly mention the slight difference between the methods from the reference and ours. In Colacio's work, a two-dimensional (2D) structure with a spin-canted antiferromagnet that led to a weak ferromagnetism was synthesized by the reaction of the metal ions and pymca ligands (molar ratio of 1 : 1) in a water solution at 190 °C.

**Scheme 2:**
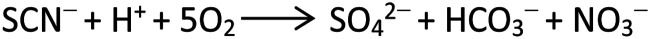
The oxidation process for thiocyanate to the sulfate ion.

To obtain the 1D structure, a more effective method was used to reduce the coordination ability of the pymca ligand by changing the polarity of the reaction solvent. Therefore, in this study, CH_3_CN, whose polarity is weaker than that of water, was used as one of the solvents. The title complex 1 was obtained by the reaction of Co(SCN)_2_, 2-cyanopyrimidine and a H_2_O/CH_3_CN mixed solution. However, the results showed that a sulfate ion appeared in the structure instead of a thiocyanate ion. This phenomenon was attributed to the oxidation of the SCN^−^ ion to a SO_4_^2−^ ion (Scheme 2).^32^

Therefore, in order to examine the effects of different charge balancing ions (anions), the synthesis of complex 1 was performed again. The reaction conditions were the same as those mentioned above, except that Co(SCN)_2_ was replaced by CoSO_4_·7H_2_O, CoCl_2_, and Co(ClO_4_)_2_, Co(NO_3_)_2_, respectively. However, the crystals of complex 1 were obtained only in the CoSO_4_ solution, implying that the presence of the Cl^−^, ClO_4_^−^, NO_3_^−^ ions could not produce the title complex. The different anions herein mainly regulated the pH of the reaction system. Another unexpected result was that the pymca directly used as the reactant could not obtain 1 under the same conditions. We speculated that complex 1 needed to be crystallized in a weak alkaline environment due to the production of amino groups during the *in situ* hydrolysis reaction of CN^−^ to COO^−^. When only pymca ligands were added, the reaction system was a weak acidic condition. Therefore, the ligand was hard to remove the proton and was not suitable for crystal crystallization. The above results also showed that the synthesis reaction of complex 1 depended on many factors.

### XRD and thermal analyses

The X-ray powder diffraction analysis was performed at room temperature to check the phase purity of complex 1. As shown in Fig. S1,† the experimental PXRD patterns were in agreement with the simulated patterns, demonstrating the good phase purity of 1. To further verify the thermal stability of complex 1, a thermogravimetric curve was generated (Fig. S2†). The weight loss of 6.5% was due to the loss of two lattice water molecules from 60 °C to 100 °C (calcd: 6.4%). After 120 °C, the weight loss of 12.5% corresponded to the loss of four coordinated water molecules (calcd: 12.7%). The framework started to decompose above 350 °C, finally generating the thermally stable powder. In addition, the XRD analysis showed that the crystallinity of 1 still remained at 350 °C (Fig. S3†).

### Descriptions of crystal structures

**Fig. 1:**
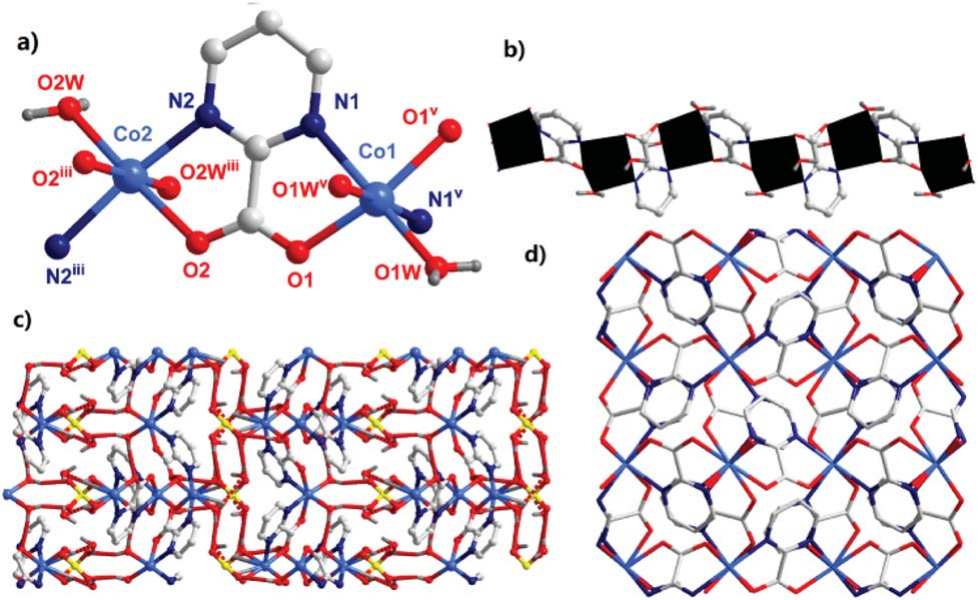
(a) The bonding environment of the Co^II^ ions (symmetry codes: (i) −*x* + 1, −*y*, *z*; (ii) −*x* + 1, −*y* + 1, *z*; w: water molecules). (b) The 1D chain was formed by dinuclear units *via* a regular alternating pattern. (c) The hydrogen bonding between the adjacent 1D chains (hydrogen bonds are represented by dashed lines). (d) The perpendicular 1D chains extend along the *a* and *b* directions and form a tetragonal grid.

The single-crystal X-ray diffraction analysis showed that complex 1 crystallized in the tetragonal system with a *P*4̄2_1_*c* space group. The asymmetric unit of 1 contained two Co^II^ ions, two pymca ligands, four coordination water molecules (O1W, O1W^v^, and O2W, O2W^iii^), one lattice sulfate ion, and two lattice water molecules (Fig. 1a with symmetry codes). The Co1 and Co2 ions had the same coordination environment. The Co1 ion was coordinated by the N1 and O1 atoms from the same pymca ligand, N1^v^ and O1^v^ of another pymca ligand (Co1–O1 = 2.107(1) and Co1–N1 = 2.157(8) Å), and two O atoms (O1W, O1W^v^) from two coordinated water molecules, presenting a distorted octahedral geometry. Co2 was centrosymmetric and surrounded by two O atoms (O2W, O2W^iii^) from two coordinated water molecules, two carboxylate O atoms (O2, O2^iii^), and two N atoms (N2, N2^iii^) from two different pymca ligands.

Although the coordination environments of Co1 and Co2 were the same, the shapes of the distorted octahedrons were different (N1–Co1–N1^v^ = 99.3(3)°, N2–Co2–N2^iii^ = 164.9(3)°) (Fig. S4†), which meant that the neighboring two pymca ligands were not coplanar and almost vertical to each other. Their dihedral angles were 83.577° and 80.473° around Co1 and Co2, respectively. For 1, the oxygen and nitrogen atoms of the pymca ligands adopted chelated and bridged modes to connect the Co^II^ ions, forming a 1D zigzag chain (Fig. 1b), which was further packed into a 3D supramolecular structure by hydrogen bonding interactions between adjacent chains, lattice water molecules, and sulfate ions (Fig. 1c and Table S3†). The adjacent chains were perpendicular to each other and extended along *a* and *b* directions to form the final 3D supramolecular framework (Fig. 1d). The shortest Co⋯Co intrachain and interchain separations were 5.591 Å and 5.298 Å, respectively.

### DC magnetic measurements

**Fig. 2:**
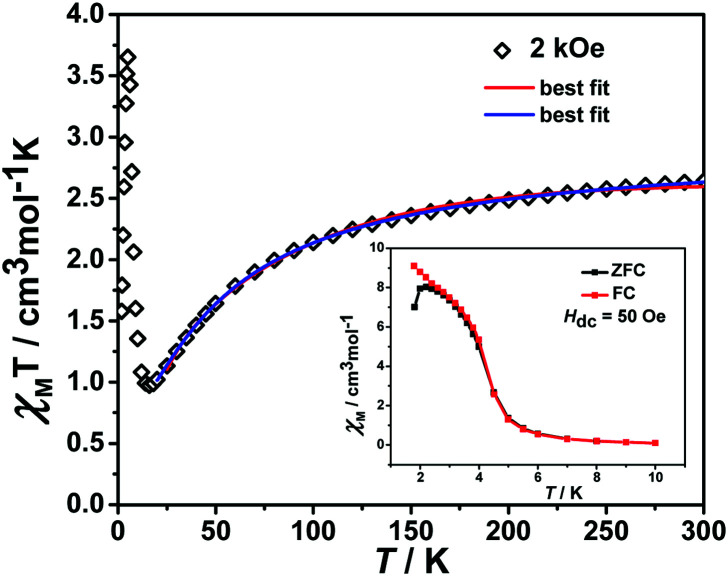
The plot of *χ*_M_*T vs. T*. The data was measured under a 2 kOe dc field. The red line is fitted using a modified empirical expression and the blue line is fitted by the phenomenological method. Insert: plot of ZFC–FC under a DC field of 50 Oe.

The variable temperature magnetic susceptibility of 1 was measured on an applied field of 2 kOe at 1.8–300 K. The *χ*_M_*T* value of 2.64 cm^3^ mol^−1^ K was larger than the theoretical value of one isolated high spin Co^II^ ion (1.875 cm^3^ mol^−1^ K with *g* = 2 and *S* = 3/2) at an ambient temperature (Fig. 2), indicating the existence of the orbital contribution of Co^II^ in the octahedral coordination ligand field.^33^ As the temperature decreased, the *χ*_M_*T* value steadily declined until 16 K and reached a minimum value of 0.95 cm^3^ mol^−1^ K, which arose from the spin–orbit coupling effects or the antiferromagnetic interactions between the Co^II^ ions.^28^ Then, the *χ*_M_*T* value rapidly increased to a maximum of 3.65 cm^3^ mol^−1^ K at 5 K and sharply decreased to 3.16 cm^3^ mol^−1^ K at 1.8 K. For one non-interacting Co^II^ ion, the limit value was 1.7 cm^3^ mol^−1^ K (ref. 34) as obtained from
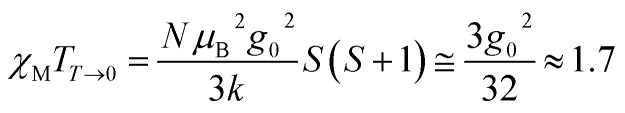
the minimum *χ*_M_*T* value of complex 1 was 0.95 cm^3^ mol^−1^ K that was lower than the limit value. Therefore, the coupling between the Co^II^ ions was antiferromagnetic. However, in the whole temperature range, *χ*_M_*T*–*T* showed a ferrimagnet-like plot instead of an antiferromagnetic curve, which implied that the canted spins possibly dominated the magnetic properties^35*a*^ because the two Co^II^ ions in the asymmetrical unit and the net spins led to a saturated *χ*_M_ value along the external field of 2 kOe. This phenomenon prompted us to further explore the SCM behavior. The field-cooling (FC) and zero-field-cooling (ZFC) were tested under a 50 Oe DC field in “Temperature Stable” mode from 1.8 K to 10 K. The ZFC and FC curves diverged at *ca.* 3 K (Fig. 2 inset), which was considered as the blocking temperature of the chain complex. From the ZFC–FC curve, the Co chain had weak ferromagnetic ordering at low temperatures. No proper models could fit the variable-temperature magnetic data in the whole temperature region. To further quantitatively interpret the behavior, a modified empirical expression^34^ for octahedral high-spin Co^II^ was employed in the temperature range of 24–300 K by replacing the *g* factor with the *G*(*T*, *J*) function, taking into account the spin-orbital coupling and exchange coupling.
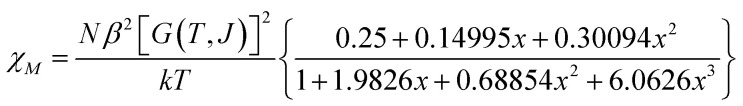
With
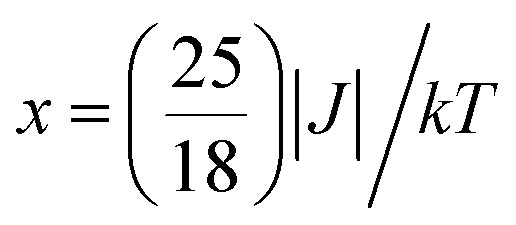
Using this model, the best-fit (red line) parameters were obtained: *J* = −6.0(3) cm^−1^, *α* = 1.18(3), *Δ* = −792(27) cm^−1^, and *λ* = −170 cm^−1^ (fixed). The negative *J* value indicated the antiferromagnetic coupling between the Co^II^ ions within the chain.

In order to further obtain the interchain interactions, the variable-temperature magnetic data was fitted by the phenomenological method (blue line) reported by Rueff *et al.*^35*b*,35*c*^ with the equation taking the mean-field correction into account as the following:*χT* = *A* exp(−*E*_1_/*kT*) + *B* exp(−*E*_2_/*kT*)*J* = −2*E*_2_/*k*
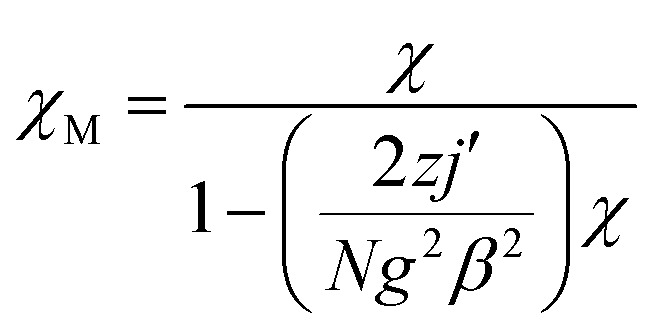
giving the Curie constant, *C* = *A* + *B* = 5.90 cm^3^ mol^−1^ K, which was consistent with six-coordinated high-spin Co^II^ ions. The spin–orbit coupling constant was −*E*_1_/*k* = −50.51 K. The spin–spin coupling constant was −*E*_2_/*k* = −2.11 K, *J* = −4.22 K (or 6.08 cm^−1^) and the interchain interaction was *zj*′ = 0.01 K using the mean-field theory correction. After comparing the two fitting results, it was found that the values of the intra-chain coupling were the same, which proved the correctness of the fitting method.

**Fig. 3:**
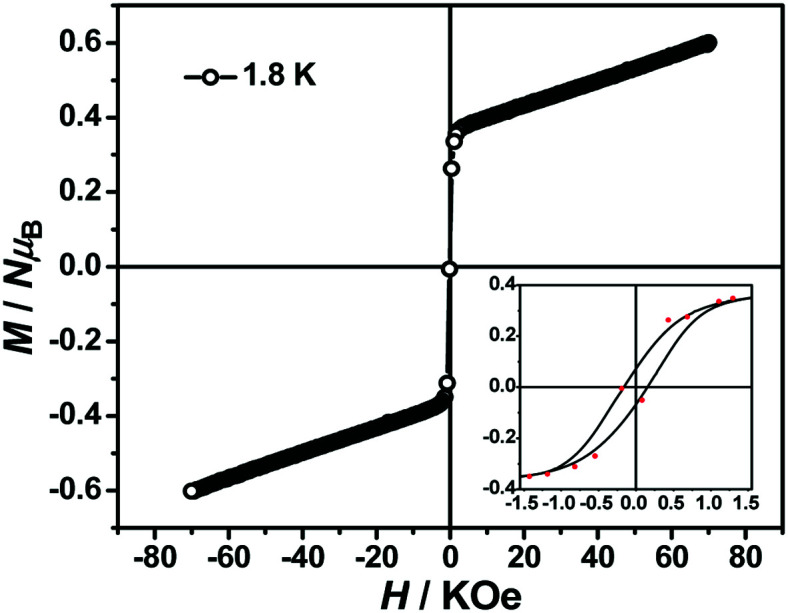
Magnetic hysteresis loops obtained at 1.8 K.

**Fig. 4:**
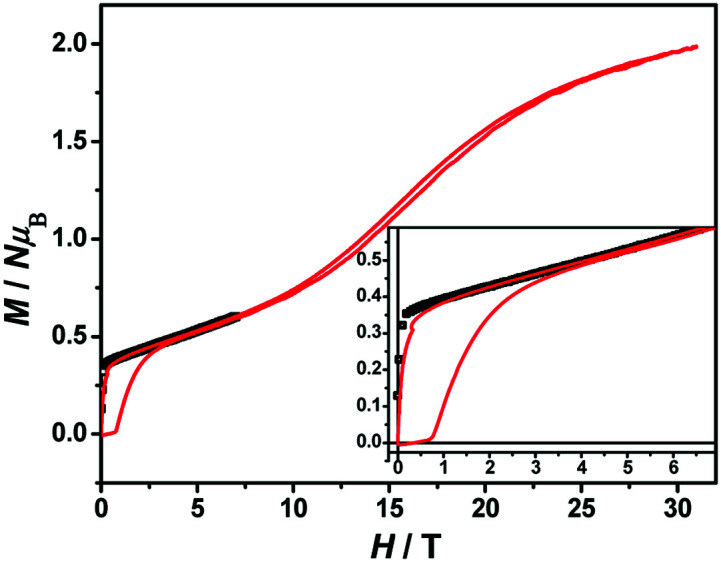
The *M*–*H* curve of 1 measured in a high field at 1.9 K.

The magnetization hysteresis loops were measured and are shown in Fig. 3 with two obvious regions at 1.8 K. In the low DC fields below 2.89 kOe, the magnetization sharply increased to 0.37 *Nμ*_B_. Above this field, the magnetization slowly increased linearly to a maximum of 0.60 *Nμ*_B_ in 7 T. For the octahedral high-spin Co^II^ at low temperatures, an effective spin with *S* = 1/2 and *g*_av_ = 4.4 was assumed for the low-lying doublet. Therefore, the saturated value for one isolated Co^II^ ion was 2.2 *Nμ*_B_. The small experimental value (0.60 *Nμ*_B_) suggested a strong antiferromagnetic coupling interaction with spin canting in 1. To determine the spin canting, the high-field magnetization was measured. The *M*(*H*) curve was obtained by calibrating to the SQUID data at 1.9 K (Fig. 4), which illustrated the main role of the antiferromagnetic coupling. Due to the quick sweeping rate of the magnetic field (5.0 kT s^−1^ on an average), virgin magnetization was observed during the increase in the field process. In the decreasing process, the low-field magnetization coincided well with the results measured in SQUID. The virgin and normal magnetizations, which led to a loop, were easily observed in the magnets with an antiferromagnetic coupling such as metamagnets.^36*a*^ In the low field, the spins for complex 1 were easily magnetized and quickly reached a platform area, which was ascribed to the saturation state of the spins in the domain. The *M* value in the platform was much lower than 2.2 *Nμ*_B_ for one Co^II^ ion. Therefore, complex 1 was a weak ferromagnetic chain based on spin canting. Upon increasing the field, an S-shape curve was observed, which was a result of the antiferromagnetic coupling between the spins quenched by the field. Field decoupling led to all the spins gradually arranging along the direction of an external field. At 30 T, the *M* = 1.99 *Nμ*_B_ approached a saturation value of 2.2 *Nμ*_B_, which was calculated based on one Co^II^ ion. These results obviously indicated the spin canting in complex 1. When the linear part of *M*–*H* (Fig. 3) in the high field was extended, the intersection point with a longitudinal axis was 0.364 *Nμ*_B_, which was roughly regarded as the fully magnetized canting spins in the chain. Therefore, the canting angle was estimated as 9.5° (sin *θ* = 0.364/2.2).

### AC magnetic measurements

**Fig. 5:**
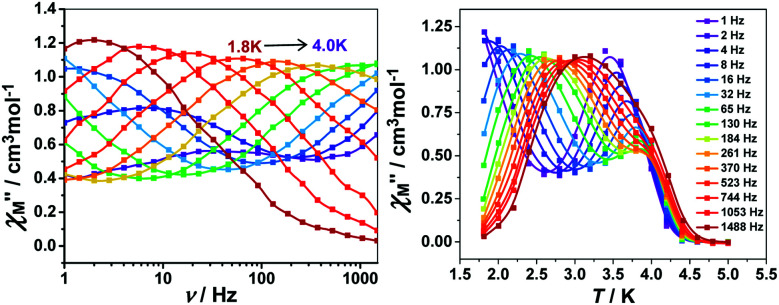
Frequency dependence of the out-of-phase (left) and temperature dependence for the out-of-phase (right) components for the AC susceptibility in 1 with a zero applied static field and an oscillating field of 5 Oe at a frequency region of 1–1488 Hz.

To further investigate the SCM properties and magnetic relaxation mechanism of complex 1, the fully ground 1 was subjected to alternating current (ac) magnetic measurements from 1 Hz to 1488 Hz at lower temperatures of 1.8 K-5.0 K. There were two obvious frequency-dependent peaks for both, the in-phase 
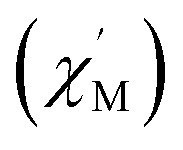
 and out-of-phase 
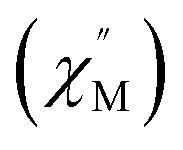
 AC susceptibility (Fig. 5 and S5†). Below 3.2 K, the maximum value for 
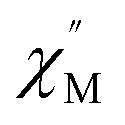
 moved to a higher temperature as the frequency increased for the 
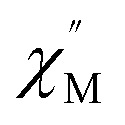
–*T* curves. The Mydosh parameter (*φ*) was estimated to be 0.29 (*φ* is used to measure the frequency dependence, *φ* = (Δ*T*_p_/*T*_p_)/Δ(log *f*),^36*b*^*T*_p_ denotes the temperature for the maximum in the *χ*′′–*T* curves, and *f* is the AC frequency), which was in the range of 0.1–0.3 as expected for the superparamagnetic behavior in SCMs. Conversely, *φ* was calculated to be 0.07 for the high-temperature region of 3.4–5.0 K, corresponding to spin glass characteristics (0.01 < *φ* < 0.08).^36*c*,36*d*,37^ A Cole–Cole curve was significantly semicircular and distributed only at 1.8–3.2 K (Fig. S6a†). In the graph we clearly observed a slow relaxation process in this temperature range. The Arrhenius plots of the relaxation times (*τ*) were obtained from the temperature-dependent 
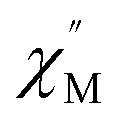
 peaks because the peaks could only be clearly separated in Fig. 5 in two temperature regions. According to the Arrhenius law, *τ* = *τ*_0_ exp(Δ*τ*/*k*_B_*T*), the anisotropy barrier (Δ*τ*/*k*_B_) and the pre-exponential factor (*τ*_0_) were extracted, giving a value of Δ*τ*/*k*_B_ = 31.2 K, which was close to that in previously reported literature.^38–40^ The values of *τ*_0_ = 5.4 × 10^−9^ s (2.0–3.2 K) and 152.4 K, 1.5 × 10^−20^ s (3.4–5.0 K), respectively (Fig. S6†) exceeded that in literature.^41,42^ The long spin reversal time fell within a range for low-dimensional magnets (SCM), while the short spin was suggested from the spin glasses. The data obtained at high temperatures was fitted using the conventional critical scaling law for spin dynamics (Fig. S7†). According to the equation, *τ* = *τ*_0_[(*T*_p_ − *T*_f_)/*T*_f_] − *zν*, the parameters were *τ*_0_ = 1.32 × 10^−4^ s, *zν* = 12, and *T*_f_ = 2.2 K. The *zν* value fell in the region for all kinds of spin glasses (4–12), and the frozen temperature was 2.2 K.^37,43,44^ The high-temperature signal observed in the out-of-phase indicated the formation of magnetic domains due to the interchain ferromagnetic interactions with a short distance between chains. The examples of weak ferromagnetic intermolecular interactions that lead to 3D magnetic properties are very rare because the antiferromagnetic intermolecular interactions always make the system more stable.^43^

**Fig. 6:**
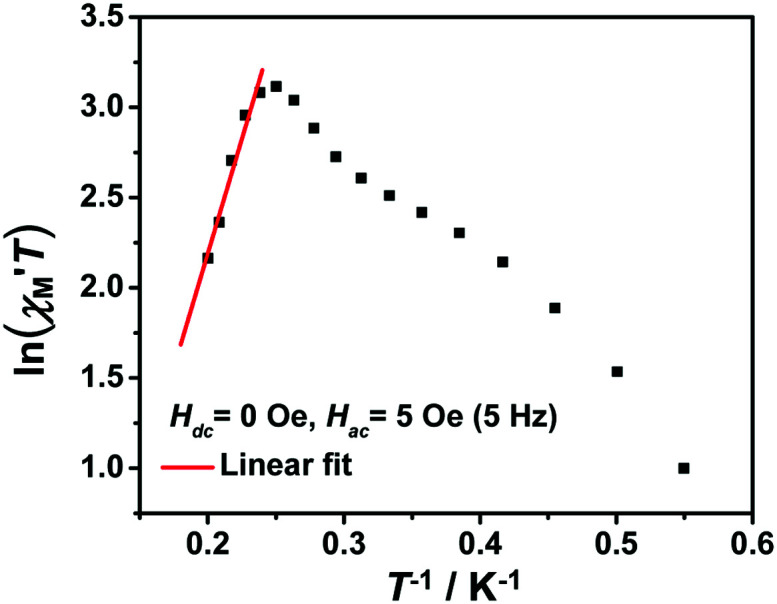
The 
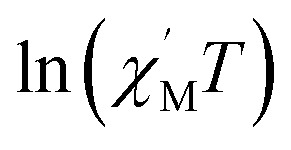
*vs.* 1/*T* curve of 1 collected in a DC field of 0 Oe on an oscillating field of 5 Oe at 5 Hz. The solid red line represents the fit of the linear part of the data.

The variable-temperature magnetic susceptibility (*χ*_M_*T*) was proportional to the correlation length (*ξ*) in any 1D typical system under a DC field of 0 Oe. For an Ising-like or anisotropic Heisenberg 1D system, as the temperature decreases, *χ*_M_*T* increases exponentially, which can be expressed by the following equation: *χ*_M_*T* ≈ *C*_eff_ × exp(*Δ*_*ξ*_/*k*_B_*T*). The parameters in the expression, *C*_eff_ and *Δ*_*ξ*_, denote the effective Curie constant and the correlation energy used to create a domain wall along the chain, respectively.^10,45–47^ According to the above equation, as shown in Fig. 6, the plot of 
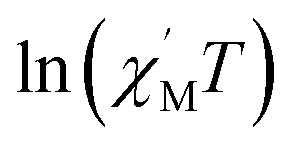
*versus* 1/*T* is a straight line, which is consistent with the above-mentioned model, whose slope is equal to the correlation at 4–5 K and yielded a *Δ*_*ξ*_/*k*_B_ = 25.36 K and *C*_eff_ = 0.056 cm^3^ mol^−1^ K. The decrease in the 
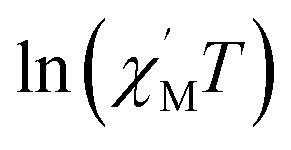
 value indicated that the correlation length was larger than the average separation between the two intrinsic defects along the chain. We noticed a broad shoulder bulge in Fig. 6, implying that the interchain interaction demonstrated some influence compared to the anisotropy of the chain.^48^ For 1D systems falling within the Ising limit, when the spin was canted along the chain, the activation energy (*Δ*_*ξ*_) was calculated by the expression *Δ*_*ξ*_ = 4|*J*|*S*^2^ cos *θ* (Co^II^ at low temperatures was an *S* = 1/2 effective spin). The calculated value was 8.52 K, which was much less than the value fitted by the plot of 
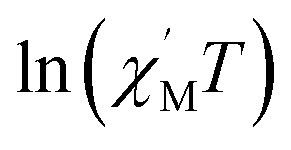
*versus* 1/*T*. This result showed that in this chain system, the relationship between *Δ*_*ξ*_ and the magnetic parameters (*S*, *J*) was uncertain.^45*b*^

From the Glauber dynamics, the energy barrier for the spin reversal should be Δ*τ* = *Δ*_*ξ*_ for finite-sized Ising chains and Δ*τ* = 2*Δ*_*ξ*_ for infinite Ising chains,^9,11,49^ wherein naturally occurring defects limit the growth of the correlation length. However, the anisotropic Heisenberg model is more common in elaborating the spin reversal energy barrier of SCMs because the anisotropy energy of each magnetic unit was considered in the model.^20^ The correlation length (*ξ*) increased exponentially when the temperature decreased. The overall energy barrier (Δ*τ*) was Δ*τ* = 2*Δ*_*ξ*_ + *Δ*_A_ for an infinite chain at high temperatures and Δ*τ* = *Δ*_*ξ*_ + *Δ*_A_ for a finite-sized chain at low temperatures, where *Δ*_A_ denotes the intrinsic anisotropic barrier for the individual spin without a magnetic exchange.^45^ According to the fitting above (Fig. S6b†), the low-temperature energy barrier (2.0–3.2 K) was Δ*τ*/*k*_B_ = 31.2 K, which was obviously larger than *Δ*_*ξ*_/*k*_B_. Therefore, the intrinsic anisotropic barrier (*Δ*_A_) for the finite-sized chain was estimated using *Δ*_A_ = Δ*τ*/*k*_B_ − *Δ*_*ξ*_/*k*_B_ to be 5.84 K.

## Conclusions

In summary, a novel chain was solvothermally synthesized by the reaction of Co(SCN)_2_ and 2-cyanopyrimidine. The DC magnetic data in 2 kOe and the magnetization in the range of 0 to 30 T showed that the ligand mediated the antiferromagnetic coupling between the intrachain Co^II^ ions, leading to a spin-canting order. The AC magnetic measurements and analysis results demonstrated that of spin glass at high temperatures. As the temperature decreased, the title complex showed a slow relaxation in the magnetization, resulting from the SCM component with an energy barrier of Δ*τ*_1_/*k*_B_ = 31.2 K and preexponential factor of *τ*_0_ = 5.4 × 10^−9^ s. The blocking temperature was *ca.* 3 K. The information in this study provides an example for designing new types of SCMs obtained by the hydrolysis of 2-cyanopyrimidine *in situ*.

## Conflicts of interest

There are no conflicts to declare.

## Supplementary Material

RA-009-C9RA05354A-s001

RA-009-C9RA05354A-s002
