# Supplementary material for: A cobalt(ii) chain based on pymca generated in situ from the hydrolysis of 2-cyanopyrimidine: spin canting and magnetic relaxation
Source: RSC Adv. 2019 Oct 2;9(53):31115–21. doi: 10.1039/c9ra05354a (PMC9072564; doi:10.1039/c9ra05354a)
Supplement: RA-009-C9RA05354A-s001 [file RA-009-C9RA05354A-s001.pdf]

Electronic Supplementary Material (ESI) for RSC Advances.
This journal is © The Royal Society of Chemistry 2019

**Electronic Supplementary Information**

# A Cobalt (II) Chain Based on Pymca Generated in Situ from the Hydrolysis of 2-Cyanopyrimidine: Spin Canting and Magnetic relaxation

*Jie Zhang,[a] Qian-Nan Zhao,[a] Feng Yang,[b] Lei Yin,[b] Miao-Miao Li,[a] Zhenxing Wang,[b] Zhongwen Ouyang,[b] Zheng-Cai Xia,*[b] and Tuo-Ping Hu*[a]*

[a] Department of Chemistry, College of Science, North University of China, Xueyuan Road 3, Taiyuan 030051, PR China.

[b] Wuhan National High Magnetic Field Center & School of Physics, Huazhong University of Science and Technology, Wuhan 430074, PR China

**Table S1.** Crystallographic data of complex **1**

| **complex** | **1** |
|---|---|
| Empirical formula | $C_{10}H_{18}Co_2N_4O_{14}S$ |
| Formula weight | 568.20 |
| Crystal system | Tetragonal |
| space group | $P\bar{4}2_1c$ |
| $a$ (Å) | 9.5941(13) |
| $b$ (Å) | 9.5941(13) |
| $c$ (Å) | 20.329(3) |
| $\alpha$ (°) | 90 |
| $\beta$ (°) | 90 |
| $\gamma$ (°) | 90 |
| $V$ (Å$^3$) | 1871.2(6) |
| $T$ (K) | 296(2) |
| $Z$ | 4 |
| $\rho_{calcd}$ (g cm$^{-3}$) | 2.017 |
| *F(000)* | 1152 |
| □ (Å) | 0.71073 |
| R(int) | 0.0568 |
| final $R_1$, □$R_2$ [$I$ > 2□($I$)] | 0.0568, 0.1387 |
| $R_1$, □$R_2$ all data | 0.0630, 0.1387 |
| Data / restraints / parameters | 2103 / 1 / 143 |
| Largest diff. Peak and hole[e·Å$^{-3}$] | 0.924 and -0.573 |
| goodness of fit, GOF | 1.071 |
| CCDC number | 1949664 |

**Table S2.** Bond lengths [Å] and angles [°] for complex **1**.

| Complex **1** | | | |
|---|---|---|---|
| Co1 -O1W$^{i}$ | 2.077(6) | Co1-N1 | 2.158(6) |
| Co1-O1W | 2.077(6) | Co1-O1$^{i}$ | 2.108(5) |
| Co1-N1$^{i}$ | 2.158(6) | Co1-O1 | 2.108(5) |
| Co2-O2$^{ii}$ | 2.119(6) | Co2-N2$^{ii}$ | 2.141(6) |
| Co2-O2W$^{ii}$ | 2.069(8) | Co2-O2 | 2.119(6) |
| Co2-O2W | 2.069(8) | Co2-N2 | 2.141(6) |
| O1-Co1-N1$^{i}$ | 86.8(2) | O1-Co1-N1 | 78.4(2) |
| O1$^{i}$-Co1-N1 | 86.8(2) | O1$^{i}$-Co1-O1 | 157.0(3) |
| O1W$^{i}$-Co1-O1W | 88.1(3) | O1W-Co1-O1$^{i}$ | 93.4(2) |
| O2W$^{ii}$-Co2-O2W | 88.7(5) | O2W-Co2-N2 | 99.8(2) |
| O2W-Co2-N2$^{ii}$ | 91.0(2) | O2-Co2-N2 | 78.3(2) |
| O2W-Co2-O2$^{ii}$ | 87.8(3) | O2-Co2-N2$^{ii}$ | 91.6(2) |
| O1W-Co1-N1 | 174.3(2) | O1W-Co1-O1 | 103.1(2) |
| O1W$^{i}$-Co1-N1 | 86.3(2) | O1W$^{i}$-Co1-O1 | 93.4(2) |
| O1W-Co1-N1$^{i}$ | 86.3(2) | N1-Co1-N1$^{i}$ | 99.3(3) |
| O1W$^{i}$-Co1-N1$^{i}$ | 174.3(2) | O1$^{i}$-Co1-N1$^{i}$ | 78.4(2) |
| O1W$^{i}$-Co1-O1$^{i}$ | 103.1(2) | O2W$^{ii}$-Co2-N2 | 91.0(2) |
| O2W$^{ii}$-Co2-O2 | 87.8(3) | O2W$^{ii}$-Co2-N2$^{ii}$ | 99.8(2) |
| O2W$^{ii}$-Co2-O2$^{ii}$ | 175.9(3) | O2-Co2-O2$^{ii}$ | 95.8(3) |
| O2W-Co2-O2 | 175.9(3) | | |

Symmetry transformations used to generate equivalent atoms: i: -x+1, -y, z, ii: -x+1, -y+1, z.

**Table S3.** Hydrogen bonds for complex **1**. Bond lengths [Å] and angles [°].

| D—H···A | d(D—H) | d(H···A) | d(D···A) | <(D—H···A) |
|---|---|---|---|---|
| O(1W)—H(1A) ···O(4) | 0.85 | 1.91 | 2.735(9) | 164 |
| O(1W)—H(1B) ···O(2) | 0.85 | 1.96 | 2.793(8) | 168 |
| O(2W)—H(2A) ···O(3) | 0.87 | 2.06 | 2.913(12) | 168 |
| O(2W)—H(2B) ···O(3W) | 0.74 | 2.34 | 2.961(14) | 143 |
| O(3W)—H(10A)···O(4) | 0.85 | 2.16 | 2.847(16) | 137 |
| O(3W)—H(10B) ···O(3) | 0.85 | 2.40 | 2.683(12) | 100 |

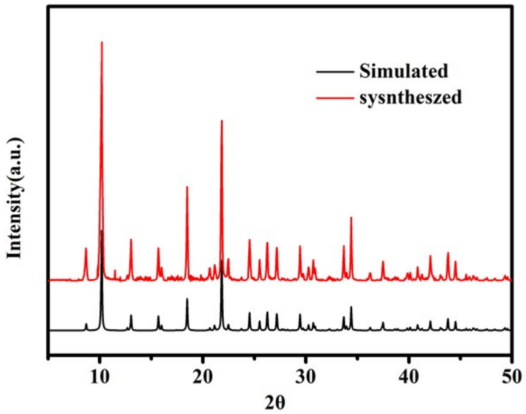


**Fig. S1** The PXRD pattern of **1.**

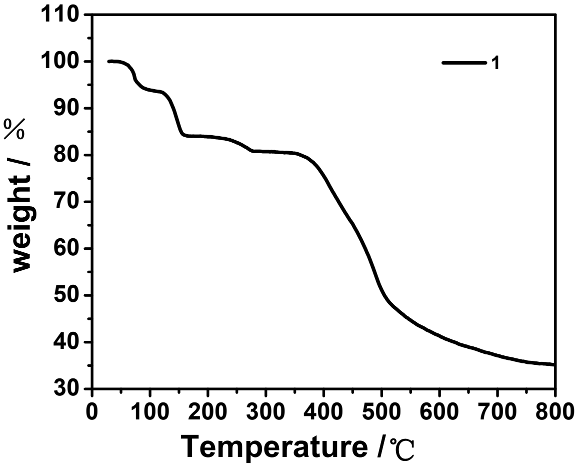


**Fig. S2** The TG curves of complex **1.**

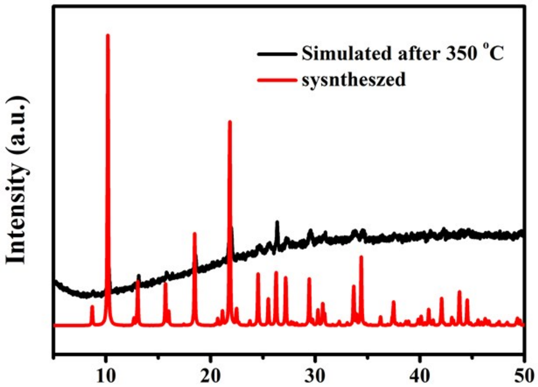


Fig. S3 The PXRD pattern of the **1** heated to 350 °C.

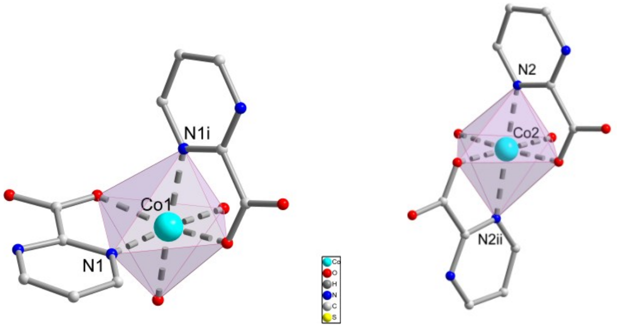


**Fig. S4** The distorted octahedral coordination geometry of Co1 and Co2 (N1-Co1-N1i = 99.3(3)° (left), N2-Co2-N2i = 164.9(3)°) (right).

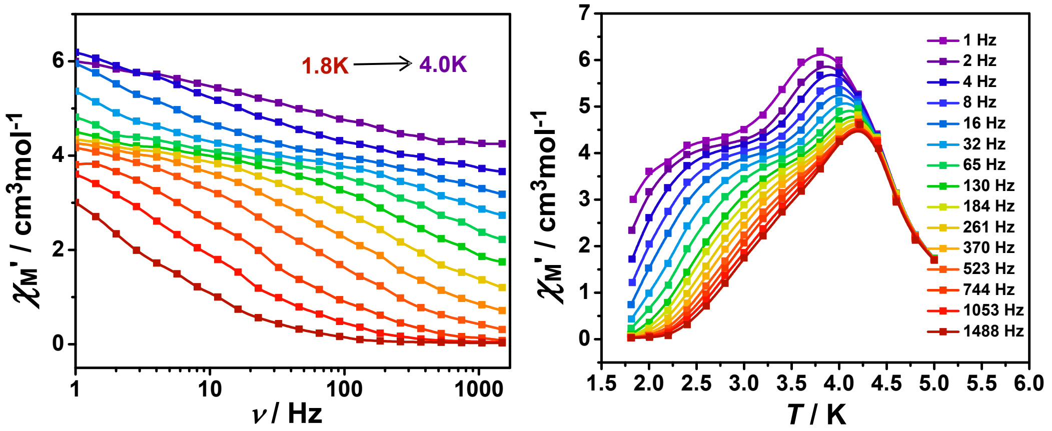


**Fig. S5** Frequency dependence of in-phase (left) and temperature dependence of in-phase (right) components of the ac susceptibility for the complex **1** in zero applied static field with a 5 Oe oscillating field at a frequency of 1–1488 Hz.

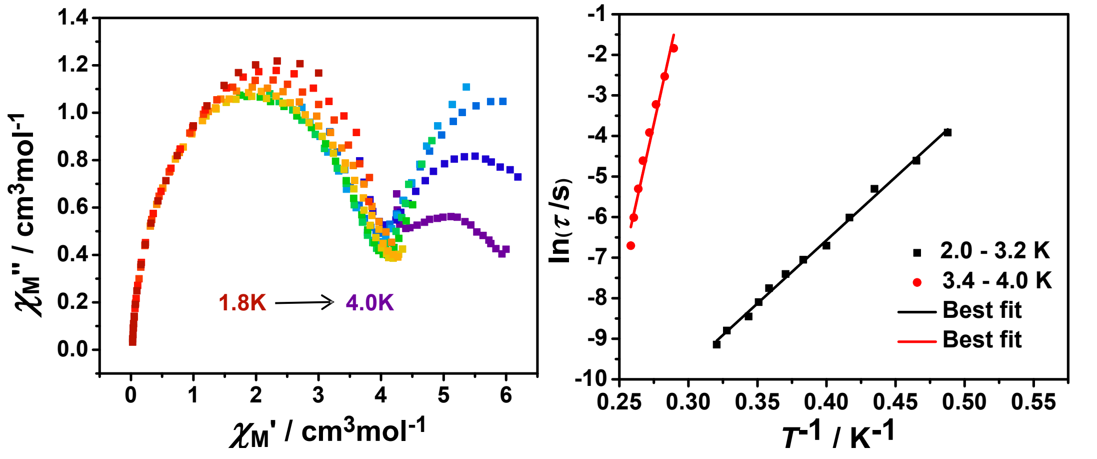


**Fig. S6** (a) A plot of cole-cole curve, (b) the plot of ln$\tau$ vs. 1/$T$.

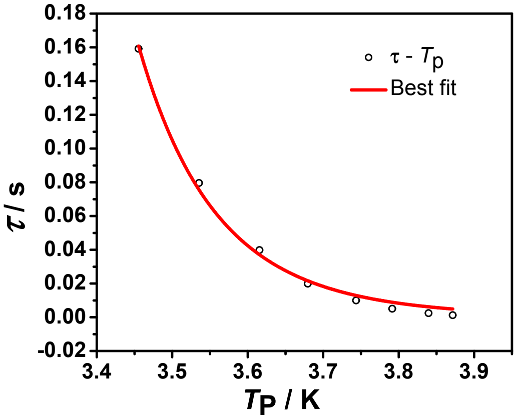


**Fig. S7** The plot of $\tau$ vs. $T$p for **1** was fitted by the conventional critical scaling law of the spin dynamics as described by $\tau = \tau_0\,[(T_P - T_f)/T_f]^{-z\nu}$.
